# Repeated evaluations of the quality of clinical teaching by residents

**DOI:** 10.1007/s40037-013-0060-5

**Published:** 2013-05-08

**Authors:** Cornelia R. M. G. Fluit, Remco Feskens, Sanneke Bolhuis, Richard Grol, Michel Wensing, Roland Laan

**Affiliations:** 1 Academic Educational Institute, Radboud University Nijmegen Medical Centre, 306 IWOO, PO Box 9101, 6500 HB Nijmegen, the Netherlands; 2 Scientific Institute for Quality of Healthcare, Radboud University Nijmegen Medical Centre, 306 IWOO, PO Box 9101, 6500 HB Nijmegen, the Netherlands; 3 Department of Rheumatology, Radboud University Nijmegen Medical Centre, 306 IWOO, PO Box 9101, 6500 HB Nijmegen, the Netherlands; 4 Department of Methods and Statistics, Utrecht University, Utrecht, the Netherlands

**Keywords:** Clinical teacher, Evaluation, Assessment, Instrument, Postgraduate medical education

## Abstract

Many studies report on the validation of instruments for facilitating feedback to clinical supervisors. There is mixed evidence whether evaluations lead to more effective teaching and higher ratings. We assessed changes in resident ratings after an evaluation and feedback session with their supervisors. Supervisors of three medical specialities were evaluated, using a validated instrument (EFFECT). Mean overall scores (MOS) and mean scale scores were calculated and compared using paired T-tests. 24 Supervisors from three departments were evaluated at two subsequent years. MOS increased from 4.36 to 4.49. The MOS of two scales showed an increase >0.2: ‘teaching methodology’ (4.34–4.55), and ‘assessment’ (4.11–4.39). Supervisors with an MOS <4.0 at year 1 (*n* = 5) all demonstrated a strong increase in the MOS (mean overall increase 0.50, range 0.34–0.64). Four supervisors with an MOS between 4.0 and 4.5 (*n* = 6) demonstrated an increase >0.2 in their MOS (mean overall increase 0.21, range −0.15 to 53). One supervisor with an MOS >4.5 (*n* = 13) demonstrated an increase >0.02 in the MOS, two demonstrated a decrease >0.2 (mean overall increase −0.06, range −0.42 to 0.42). EFFECT-S was associated with a positive change in residents’ ratings of their supervisors, predominantly in supervisors with relatively low initial scores.

## Introduction

Physicians play a crucial role in teaching residents in clinical practice [1]. They can be supported in doing so effectively by evaluating and providing feedback on their clinical teaching performance [2]. The most prevalent evaluation methodology is the completion of a standardized teacher-rating form by learners [1–3]. There are many instruments for providing feedback to clinical teachers, often based on roles that are defined in the literature about good clinical teaching [1, 4–12]. Several authors have expressed concerns about many of these instruments as they do not cover all important aspects of clinical teaching, lack a clear theoretical framework and/or have insufficient validity evidence [13–16]. This can make it difficult to establish directions in which efforts for improving teaching should be headed and to accomplish real improvement [14, 16].

Studies, focusing on the effects of evaluating clinical teachers, demonstrate an increase in clinical teaching scores after written feedback, whereas feedback in the form of simple numerical rating scores has not improved teaching scores [17]. Reading an analysis of clinical teaching or being aware of weaknesses does not automatically invite teachers to improve their teaching practice [18]. To further enhance this reflection and decision-making, discussion of the feedback with a facilitator (e.g., a peer, head or resident) seems useful [18, 19].

In 2009, our hospital started to evaluate our clinical teachers in postgraduate medical education with the EFFECT (Evaluation and Feedback For Effective Clinical Teaching) questionnaire. Different sources of validity evidence have been collected and a carefully designed system for using EFFECT in practice was developed [3, 20]. As evaluating clinical teachers is time consuming for both clinical teachers and residents, we wanted to know whether changes appear in clinical teachers’ ratings after an evaluation and feedback session using EFFECT. We sought answers to the following questions: (1) did resident ratings of clinical teachers improve after our feedback strategy, and (2) was the degree of improvement related to the initial rating?

## Methods

### Setting and study population

A prospective pre-post test study was conducted in three medical specialities in a major university medical centre in the Netherlands in 2009–2011. The study was designed as a pilot to inform the design of a larger evaluation. Residents were invited by e-mail to evaluate their supervisors by filling in a questionnaire and were free to choose which and how many supervisors they wanted to evaluate. Supervisors filled in a self-evaluation form. Evaluations took place in two subsequent years. The evaluation results were discussed during a face-to-face meeting between the supervisor and two residents or the head of department [3].

### The EFFECT questionnaire

The EFFECT questionnaire is a validated questionnaire based on literature on clinical teaching in the workplace and incorporates the CanMEDS competencies. EFFECT contains 58 items grouped into eleven subscales: (1) role modelling clinical skills, (2) role modelling science, (3) role modelling CanMEDS competencies, (4) role modelling reflection, (5) assigning work relevant for learning, (6) planning, (7) feedback process, (8) feedback content, (9) teaching methodology, (10) assessment, and (11) personal support. Items can be scored on a five-point Likert scale (1 = very poor, 2 = poor, 3 = intermediate, 4 = satisfactory, 5 = good). Details of the instrument, the validation process, and the evaluation system are described elsewhere [3].

### The system for evaluation and feedback for effective clinical teaching (EFFECT-S)

EFFECT-S starts with an introduction meeting with staff and residents at the department to inform (and involve) them about the formative purpose of the evaluation procedure in their department and to make tailor-made appointments. A tailor-made EFFECT-S includes a careful planning; a discipline-specific questionnaire; agreement on who fills in the questionnaires (residents on a voluntary basis, anonymous ratings), how the feedback procedure is organised, and who has access to the results. The evaluation itself consists of (a) an internet-based self-evaluation questionnaire for supervisors and a questionnaire to be completed by residents, (b) a feedback report, including the mean scores per item and domain, a group score (the mean scores of all staff of the particular department) and the written comments, and (c) a face-to-face meeting (dialogue) between the supervisor and two residents (representing their group) and guided by a moderator (an experienced educationalist) from outside the department. In one department, these meetings were done by the programme director [20].

### Analyses

Mean overall scores (MOS, mean score of all items of EFFECT) and mean scores on the separate subscales of EFFECT at year 1 and year 2 were calculated. We performed paired T-tests to test differences between the two consecutive measurements. To assess relevance of changes we translated the scores into effect sizes. As this was the first follow-up evaluation of EFFECT, we did not perform statistical power analysis.

### Ethics

The ethics committee waived approval. Participation was voluntary for departments. Confidentiality was ensured. Supervisors and residents of the participating departments were informed about the EFFECT questionnaire by the head of the department and/or during the initial meeting. The EFFECT questionnaire was filled in anonymously by residents.

## Results

Twenty-four supervisors from three departments were evaluated by three or more residents in two subsequent years. A total of 237 questionnaires were obtained. Paired T-tests showed a significant increase in the teaching methodology scale (from 4.34 to 4.55) (*p* < 0.05). Relevant improvement on EFFECT was defined as at least a moderate effect size of 0.4, which translated into an absolute change of at least 0.2 on the EFFECT MOS and subscales. Relevant improvements were found on the teaching methodology (from 4.34 to 4.55) and the assessment scale (from 4.11 to 4.39).

Table 1 shows that the five supervisors with an MOS <4.0 in year 1 all demonstrated a relevant increase in the MOS after 1 year (mean increase 0.50, range 0.34–0.64); the number of subscales within this group demonstrating a relevant increase varied from five to ten.

**Table 1:** Improvement of mean overall score for low and high scoring supervisors

Supervisors with	Number of supervisors	Mean overall improvement	Mean number of subscales improved	Mean number of subscales declined	Number of supervisors with increased score >0.20	Number of supervisors with decreased score >0.20
Mean overall score <4.0	*n* = 5	+0.50	7.4	–	5 (100 %)	–
Mean overall score 4.0–4.5	*n* = 6	+0.21	5	0.8	3 (50 %)	–
Mean overall score >4.5	*n* = 13	−0.06	1.5	2.8	1 (8 %)	2

Only supervisors with at least three evaluations at year 1 and year 2 were included

Four supervisors with an MOS between 4.0 and 4.5 (*n* = 6) demonstrated a relevant increase in their MOS of >0.2 (mean increase 0.21, range −0.15–0.53). Of the 13 supervisors with a high MOS (>4.5), one demonstrated a further increase in the MOS of >0.2, whereas two supervisors showed a decrease in their MOS of >0.2 (mean increase −0.06, range −0.42–0.42).

**Fig. 1:**
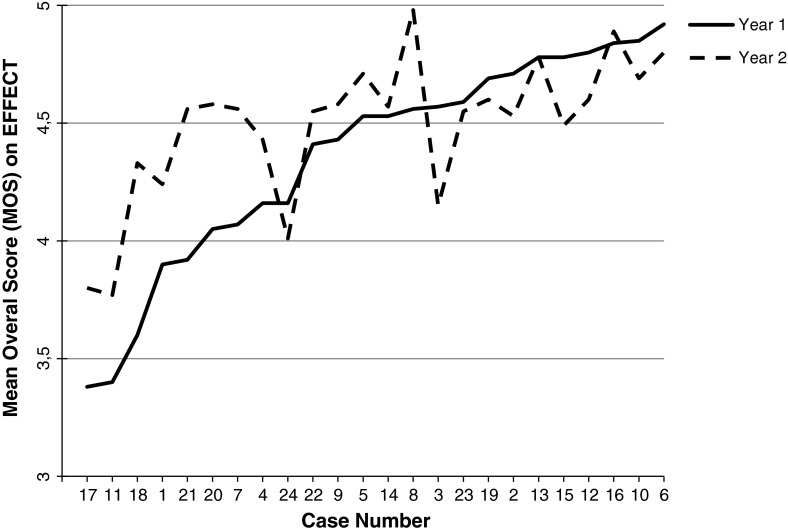
Mean overall scores on EFFECT per supervisor (*n* = 24) on two subsequent years (supervisors were evaluated by at least three residents on both measurements)

Overall scores of the 24 supervisors are shown in Fig. 1, starting with the lowest scoring supervisor at year 1. The two supervisors with the lowest MOS (3.38 and 3.40) at year 1 improved their MOS the most, although their scores at year 2 remained below 4.0 (3.80 and 3.77, respectively). The other three supervisors with an MOS < 4.0 at year 1 succeeded in increasing their MOS to over 4.0.

## Discussion

EFFECT-S was associated with relevant improvements in residents’ ratings of their supervisors on the EFFECT questionnaire. Our results indicate that those who needed to improve most do so after an evaluation and feedback session. This is in line with other research where they found that baseline compliance was the only factor that helped to explain variation in absolute effectiveness [21]. The greatest increase was found in the teaching methodology and assessment scales. The behaviours encompassed by these scales are reported to be highly beneficial for teaching effectiveness in clinical practice [22]. On the other hand, supervisors who scored high at year 1 showed little further improvement. This may be due to a ceiling effect in the measure or in what can be achieved realistically. In addition, residents may have became more critical over time. The result was that the contrast between the best and worst supervisors was reduced.

The strong asset of this study is that effects were measured with a validated instrument and did not rely on teachers self-perceptions of their improvements or on non-validated instruments [2, 17]. Our study was not designed to attribute changes to the feedback, as a control group was lacking. One serious threat of this study is that the changes we measured over time could be due to regression to the mean (RTM) [23]. However, supervisors were evaluated by at least three residents at both measurements, and it is very unlikely that the residents at both measurements are the same. Furthermore, residents were not informed about previous results. Therefore our results are probably less affected by measurement errors, but this cannot be ruled out.

Further research is needed with larger samples to confirm these results and to investigate how this feedback aids supervisors in improving their teaching within the different EFFECT scales and what additional measurements could help them. Qualitative research could explore why some supervisors demonstrate a decrease in their teaching qualities. To further optimize improvements, we need a better understanding of the effective ingredients of EFFECT-S, and the various contextual factors influencing supervisors’ behaviour.

## Essentials

- The EFFECT questionnaire covers seven domains of clinical teaching: role modelling, task allocation, planning, feedback, teaching methodology, assessment, and personal support. Behaviours are linked to the CanMEDS roles.
- EFFECT-S is a carefully designed system in which residents provide formative feedback in a dialogue with their clinical supervisor after filling in the EFFECT questionnaire.
- After participating in EFFECT-S, more than one-third of the clinical teachers show relevant improvements on the EFFECT questionnaire.
- Clinical teachers with initially low scores improve most after an evaluation and feedback session.
- Strongest improvements are in the teaching methodology and assessment domain.
